# Supplementary material for: Synthesis of fluorescent organic nano-dots and their application as efficient color conversion layers
Source: Nat Commun. 2022 Apr 4;13:1801. doi: 10.1038/s41467-022-29403-4 (PMC8980075; doi:10.1038/s41467-022-29403-4)
Supplement: Supplementary file 1 — Supplementary Information [file 41467_2022_29403_MOESM1_ESM.pdf]

Supplementary Information

Synthesis of fluorescent organic nano-dots and their application as efficient color conversion layers

Yeasin Khan, Ramanaskanda Braveenth, Soonjae Hwang, Young Hun Jung, Bright Walker*, and Jang Hyuk Kwon*

Supplementary Methods

Materials

All reagents and solvents were purchased from commercial suppliers and used without further purification. o-xylene was obtained from Samchun Pure Chemical Co., Ltd. (Seoul, Korea) and which was distilled from sodium/benzophenone before use. Dichloromethane and n-Hexane were bought from SK chemicals (Gyeonggi-do, Korea) and used without further purifications. Tetrabutyl ammonium hydroxide, Polyethylene glycol tert-octylphenyl ether (Triton X-100) was purchased from Sigma-Aldrich chemical company and used without any purification. Polyvinyl alcohol (PVA) having 83~89% degree of hydrolysis was used for film formation. Cellulose acetate dialysis tubing with a molecular weight cut-off 14,000 Dalton was purchased from Sigma-Aldrich.

5,10,15-tris(4-(4,6-diphenyl-1,3,5-triazin-2-yl)phenyl)-10,15-dihydro-5H-diindolo[3,2-a:3',2'-c]carbazole (Ttrz-DI), 1,2,3,5-Tetrakis(carbazol-9-yl)-4,6-dicyanobenzene, 2,4,5,6-Tetrakis(9H-carbazol-9-yl) isophthalonitrile (4CzIPN), 1,3,7,9-tetrakis(4-(tert-butyl)phenyl)-5,5-difluoro-10-(2-methoxyphenyl)-5H-4l4,5l4-dipyrrolo[1,2-c:2′,1′-f][1,3,2]diazaborinine (4tBuMB)[1] and, 2,12-di-tert-butyl-N,N,5,9-tetrakis(4-(tert-butyl)phenyl)-5,9-dihydro-5,9-diaza-13b-boranaphtho[3,2,1-de]anthracen-7-amine (CzDABNA)[2] were used as fluorescent organic materials.

Instrumentation

Ultraviolet-visible absorption spectra were collected using a Shimadzu UV-3600i Plus UV-VIS-NIR Spectrometer. Photoluminescence (PL) spectra were collected using a JASCO FP-8500 spectrofluorimeter. Absorbance spectra were obtained using a JASCO V-750 UV-Vis spectrometer; photoluminescence (PL) spectra and absolute photoluminescence quantum yield (PLQY) were obtained using a JASCO FP-8500 spectrofluorometer equipped with an ILF-835 integrating sphere. A wavelength of 350 nm was used to excite the materials for fluorescence and PLQY measurements. ^{1}H NMR spectra were recorded by a 400 MHz Bruker NMR spectrometer. High-resolution mass spectra were performed using JMS-700 (JEOL, Japan) Gas Chromatography-Mass spectrometer. Fluorescence microscopy was conducted by a Fluorescence microscope (Eclipse Ti, Nikon, Tokyo, Japan). Optical microscope images were taken with an Olympus BX51 microscope. Color conversion

efficiency was measured using a 6-inch integrating half-sphere with high speed LED spectrometer (LE-5400 Otsuka Electronics) and Keithley 2401 source-meter.

Synthesis of 5,10,15-tris(4-(4,6-diphenyl-1,3,5-triazin-2-yl)phenyl)-10,15-dihydro-5H-diindolo[3,2-a:3',2'-c]carbazole (Ttrz-DI)

2-(4-bromophenyl)-4,6-diphenyl-1,3,5-triazine (Trz, 8.85 mmol) and 10,15-dihydro-5H-diindolo[3,2-a:3',2'-c]carbazole (fused carbazole, 2.95 mmol) was mixed in a 3:1 molar ratio in the presence of tris(dibenzylideneacetone)dipalladium(0) ($Pd_2(dba)_3$, 0.08mmol), tri-*tert*-butylphosphine (*t*Bu_3P, 1.86 mmol), sodium *tert*-butoxide (NaO*t*Bu, 5.15mmol) and anhydrous *o*-xylene. All the reagents were taken into a two neck round bottom flask equipped with a condenser. The mixture was flushed with nitrogen and subjected to vacuum several times to create an inert atmosphere. Then, the reaction was heated with constant stirring at 135 °C for 12 hours under reflux. After reaction completion, the mixture was extracted with dichloromethane and deionized water several times. The aqueous phase was discarded and the dichloromethane layer was dried over anhydrous sodium sulphate. The mixture was then filtered and the remaining solvents were removed by rotary evaporation. The final product, 5,10,15-tris(4-(4,6-diphenyl-1,3,5-triazin-2-yl)phenyl)-10,15-dihydro-5H-diindolo[3,2-a:3',2'-c]carbazole (Ttrz-DI) was isolated by silica gel column chromatography. The final product was analyzed by ^1H NMR spectroscopy in $CDCl_3$ (Supplementary Figure 1 and 2).

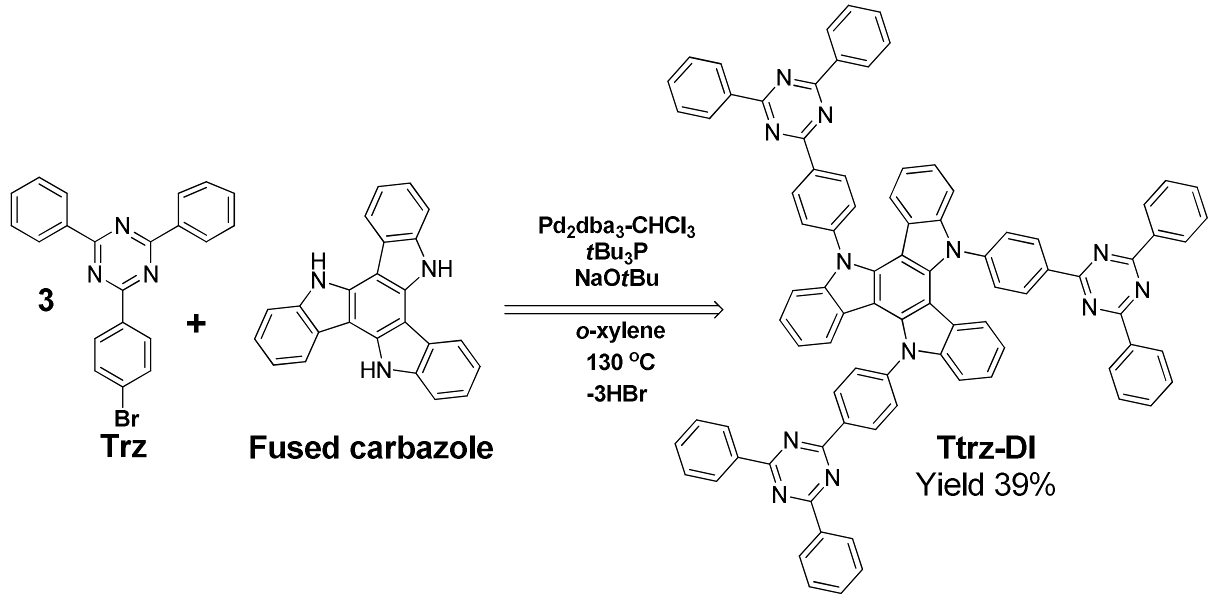

Supplementary Figure 1. Reaction scheme for the synthesis of Ttrz-DI.

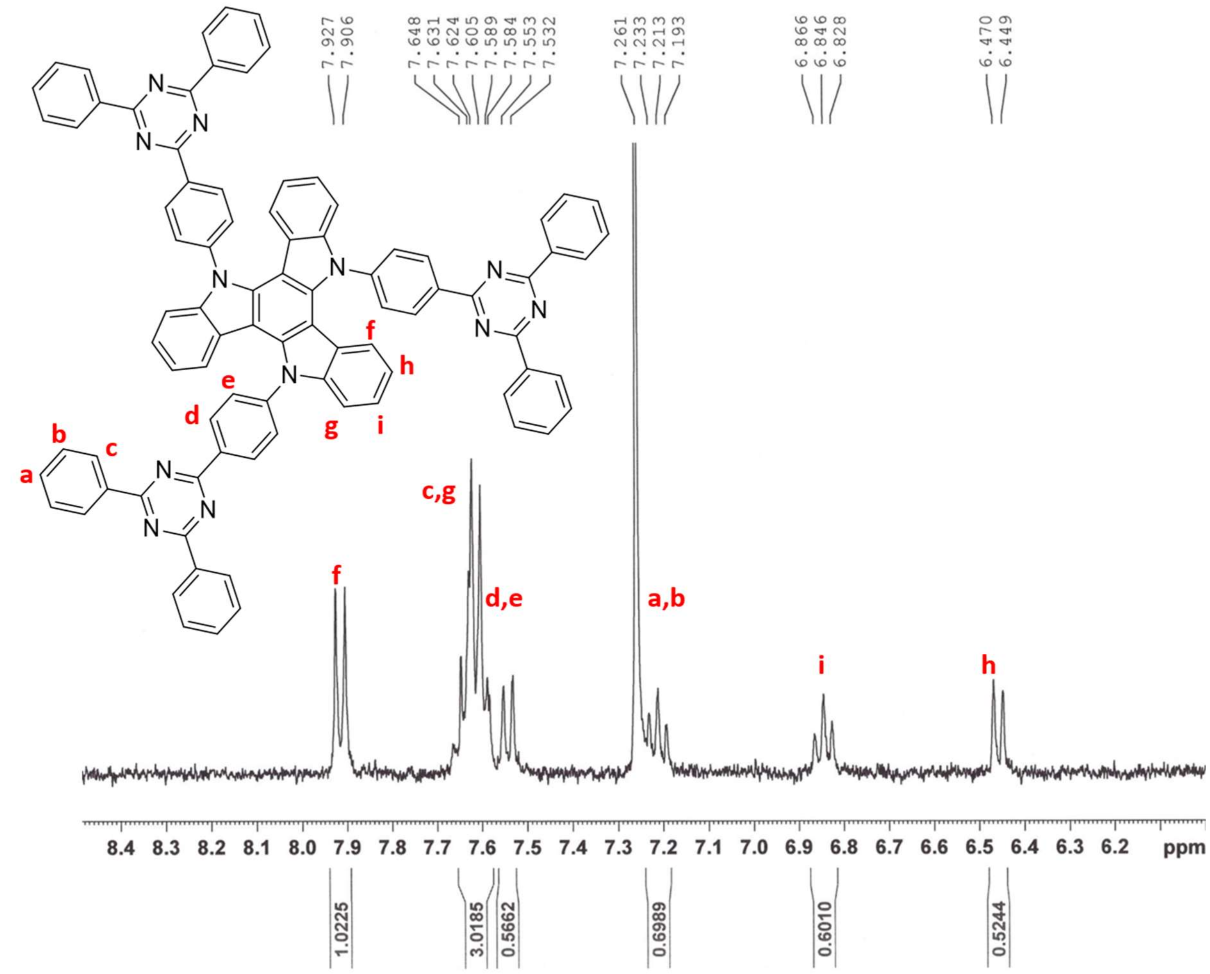

Supplementary Figure 2. ^{1}H NMR data of the synthesized molecule Ttrz-DI. Letters "a" through "i" indicate which H atom each NMR peak corresponds to in the molecular structure.

Characterization of Ttrz-DI

We first measured the basic photo physical properties of the Ttrz-DI chromophore. It showed strong absorption at around 300 nm wavelength region which corresponds to the π→π* transition of carbazole and phenyl groups with n→π* transition around 382 nm. The bandgap of the material was calculated to be 2.82 eV from the absorption onset (440 nm). Photoluminescence spectra obtained in THF solvent show a maximum at 544 nm along with solvatochromism (Supplementary Figure 3a) due to strong charge transfer characteristics.

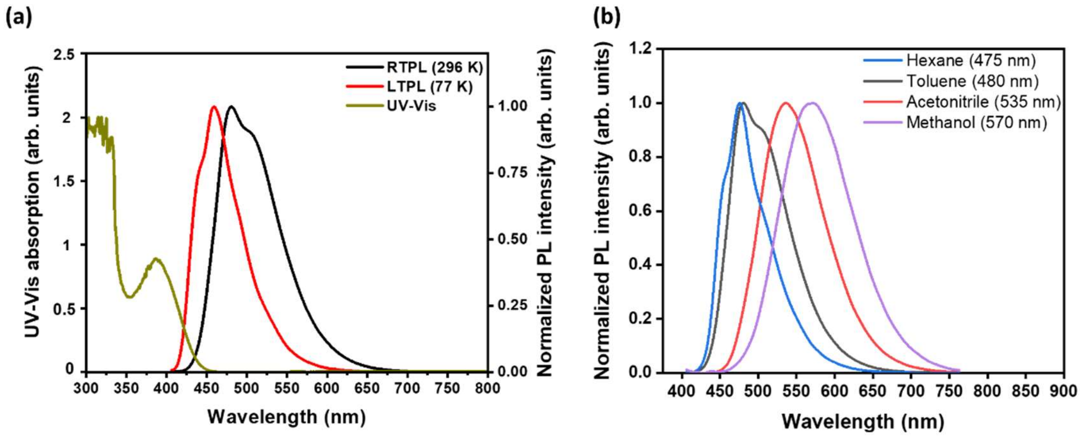

Supplementary Figure 3. Absorption and fluorescence. (a) UV-Vis, RTPL and LTPL spectra of Ttrz-DI in toluene, (b) Solvatochromism of Ttrz-DI in different solvents.

The maximum photoluminescence (PL) peak of Ttrz-DI in hexane was observed at 475 nm and the peak position was red-shifted when PL was measured in toluene, acetonitrile, tetrahydrofuran, and methanol, respectively. The onset value from the room temperature PL revealed that the singlet energy of Ttrz-DI was around 2.11 eV while a triplet energy of 2.34 eV was calculated from low-temperature PL (LTPL) analysis in toluene solvent at 77 K. The energy gap between singlet and triplet states (0.23 eV) was obtained by subtracting the triplet energy from singlet energy of Ttrz-DI (Supplementary Figure 3b).

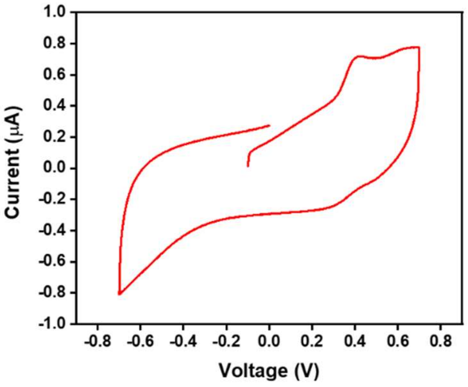

Supplementary Figure 4. Cyclic voltammogram of Ttrz-DI.

The highest occupied molecular orbital (HOMO) of Ttrz-DI was found to be -5.15 eV, calculated from its cyclic voltammogram (Supplementary Figure 4). Additionally, the respective lowest unoccupied molecular orbital (LUMO) of -2.33 eV was obtained by adding the bandgap value from UV-Vis absorption data.

Synthesis of *N,N*,6,10-tetra(naphthalen-2-yl)-6,10-dihydro-6,10-diaza-16b-boraanthra[3,2,1-*de*]tetracen-8-amine (TNAP)

1,3,5-tribromobenzene (TBB, 1 g, 3.18 mmol), di(naphthalen-2-yl)amine (NAPA, 2.74 g, 10.17 mmol), tris(dibenzylideneacetone)dipalladium(0) $Pd_2(dba)_3$ (0.26 g, 0.28 mmol), tri-tert-butylphosphine (P(t-Bu)$_3$, 0.8 mL, 3.17 mmol) and sodium tert-butoxide (NaO*t*Bu, 1.84 g, 19.15 mmol) were added in a two neck round bottom flask equipped with condenser. Then, anhydrous toluene (60 mL) was added and the mixture was refluxed at 106°C for 18 hours. After completion of the reaction, mixture was filtered and washed with hexane, then recrystallized from dichloromethane and *n*-Hexane to obtain the intermediate of N^1,N^1,N^3,N^3,N^5,N^5-hexa(naphthalen-2-yl)benzene-1,3,5-triamine (TNA) an yield of 79.35%.

Afterwards, TNA (500 mg, 0.57 mmol) was dissolved in 10 mL of *o*-dichlorobenzene at room temperature in a two neck flask equipped with reflux condenser and magnetic stirring bar. Boron tribromide BBr_3 (0.18 g, 0.74 mmol) was added drop-wise under inert conditions. Then, the reaction mixture was stirred at 180 °C for 24 hours, and the reaction progress was monitored by thin layer chromatography. After completion, reaction mixture was extracted with water and chloroform three times (150 mL), and the organic layer was dried over anhydrous sodium sulphate. After concentration under reduced pressure, the crude mixture was purified by silica gel column chromatography using *n*-hexane: dichloromethane (7:1) as a mobile phase and the product (TNAP) was collected in 23.8 % yield (Supplementary Figure 5).

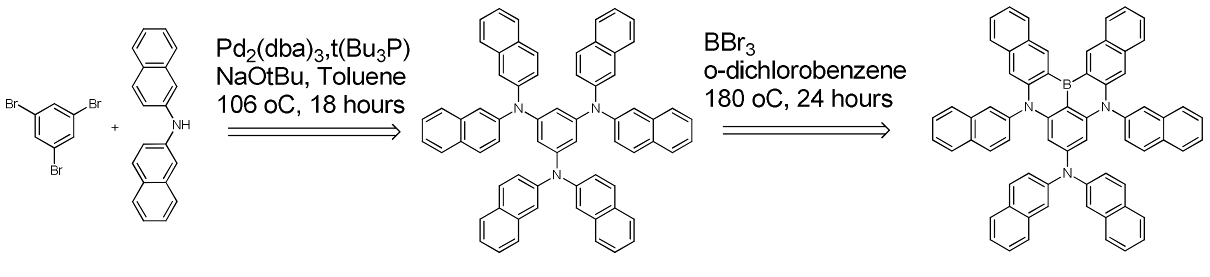

TBB **NAPA** **TNA** **Yield = 79.35%** **TNAP** **Yield = 23.8%**

Supplementary Figure 5. Reaction scheme for the synthesis of TNAP.

Characterization of TNAP

Orange color solid; ^{1}H NMR (400 MHz, CD_2Cl_2-*d*$_2$) δ (ppm) 9.75 (s, 2H), 8.23-8.26 (m, 2H), 7.79-7.85 (m, 4H), 7.69 (d, *J*=8.8 Hz, 4H), 7.52-7.55 (m, 4H), 7.42-7.49 (m, 10H), 7.34-7.41 (m, 8H), 7.30 (d, *J*=8.8 Hz, 2H), 7.19 (s, 2H), 7.05 (dd, *J*=8.0, 2.4 Hz, 2H), 5.79 (s, 2H); ^{13}C NMR (100 MHz, CD_2Cl_2-*d*$_2$) δ (ppm) 152.4, 149.2, 146.0, 143.9, 139.8, 136.4, 135.4, 134.8, 134.1, 133.0, 131.3, 130.7, 129.4, 129.1, 128.8, 128.6, 128.1, 128.0, 127.8, 127.5, 127.2, 127.1, 126.9, 126.7, 126.5, 125.3, 125.2, 123.7, 122.9, 112.5, 99.0.

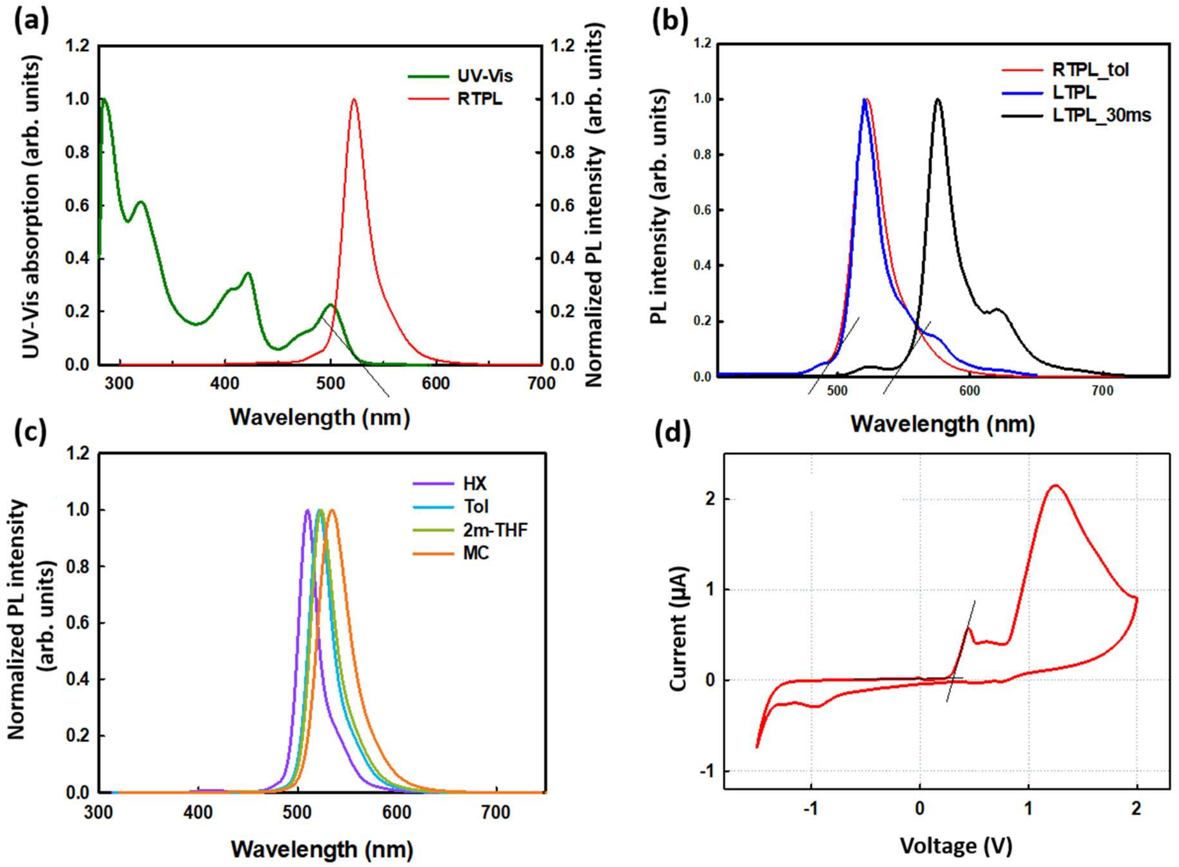

Supplementary Figure 6. Electronic characteristics. (a) UV-Vis absorption and PL emission data, (b) RTPL and LTPL spectra, (c) solvatochromism in different solvents, (d) Cyclic voltammogram of TNAP-DABNA.

The optical bandgap of TNAP was calculated to be 2.375 eV from the absorption onset (522 nm). Photoluminescence spectra obtained in THF solution showed a peak at 523 nm along with solvatochromism (Supplementary Figure 6c) due to strong charge transfer characteristics. The maximum photoluminescence (PL) peak of Ttrz-DI in hexane was obtained at 510 nm and the peak position was red-shifted when PL was measured in toluene, tetrahydrofuran, and methylene chloride respectively. The onset value from the room temperature PL revealed that the singlet energy of TNAP-DABNA is around 2.54 eV while a triplet energy of 2.28 eV was calculated from low-temperature PL (LTPL) analysis in toluene solvent at 77 K. The energy gap between singlet and triplet states (0.26 eV) was obtained by subtracting the triplet energy from singlet energy of Ttrz-DI (Supplementary Figure 6b). The HOMO of Ttrz-DI was found to be -5.43 eV calculated from its cyclic voltammogram (Supplementary Figure 6d). Additionally, the respective LUMO of -3.05 eV was obtained by adding the bandgap value from UV-Vis absorption data.

Supplementary Discussion

Characterization of nano-dots

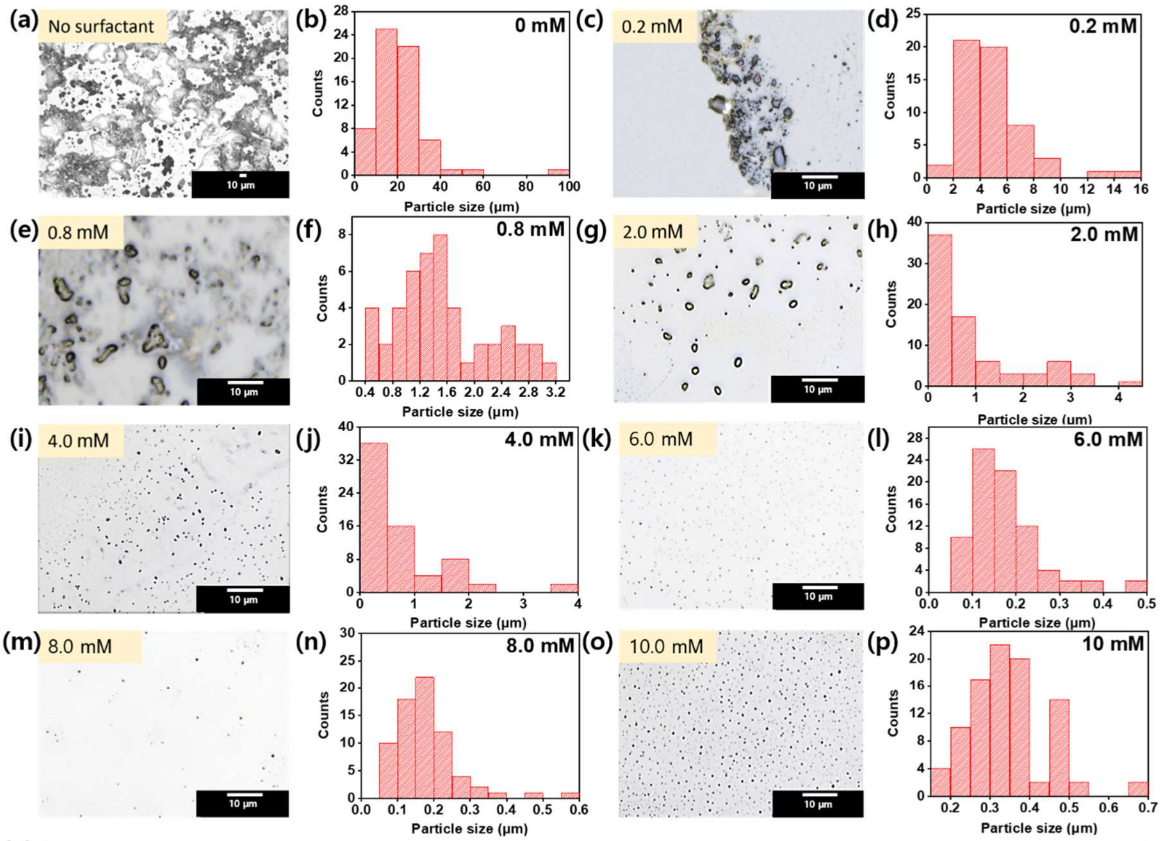

(q)

	No surfactant	0.2 mM	0.8 mM	2.0 mM	4.0 mM	6.0 mM	8.0 mM	10.0 mM
**Mean (μm)**	14.14	4.01	1.79	0.68	0.34	0.12	0.19	0.33
**SD (μm)**	10.99	1.02	0.69	0.71	0.63	0.08	0.09	0.13
**Min (μm)**	1.62	0.70	0.45	0.09	0.04	0.05	0.09	0.02
**Max (μm)**	98.22	14.71	3.57	4.29	3.57	0.48	0.58	0.69

Supplementary Figure 7. Optical images and particle distribution graphs of the Ttrz-DI nano-dots at (a, b) 0 mM, (c, d) 0.2 mM, (e, f) 0.8 mM, (g, h) 2 mM, (I, j) 4 mM, (k, l) 6 mM, (m, n) 8 mM, (o, p) 10 mM of TritonX-100 surfactant concentrations. The concentration of Ttrz-DI was kept constant at 0.01 mM; (q) Table showing their mean, standard deviation, minimum and maximum sizes.

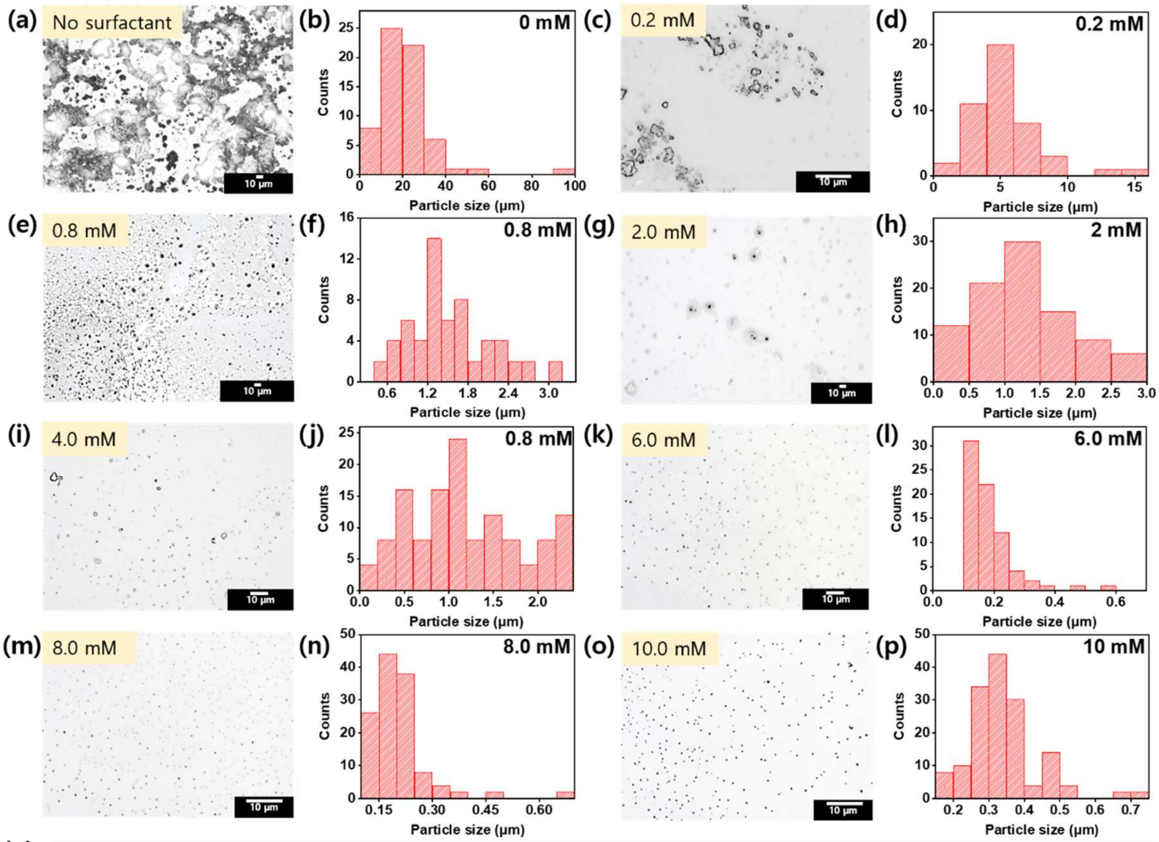

(q)

	No surfactant	0.2 mM	0.8 mM	2.0 mM	4.0 mM	6.0 mM	8.0mM	10 mM
**Mean (μm)**	14.14	5.74	1.34	1.176	0.79	0.14	0.23	0.37
**SD (μm)**	10.99	3.78	0.59	0.93	0.64	0.12	0.13	0.09
**Min (μm)**	1.62	0.54	0.34	0.11	0.18	0.11	0.12	0.17
**Max (μm)**	98.22	15.27	3.06	2.85	2.25	0.59	0.65	0.74

Supplementary Figure 8. Optical images and particle distribution graphs of the Ttrz-DI nano-dots at (a, b) 0 mM, (c, d) 0.2 mM, (e, f) 0.8 mM, (g, h) 2 mM, (I, j) 4 mM, (k, l) 6 mM, (m, n) 8 mM, (o, p) 10 mM of TBA oleate surfactant concentrations. The concentration of Ttrz-DI was kept constant at 0.01 mM; (q) Table showing their mean, standard deviation, minimum and maximum sizes.

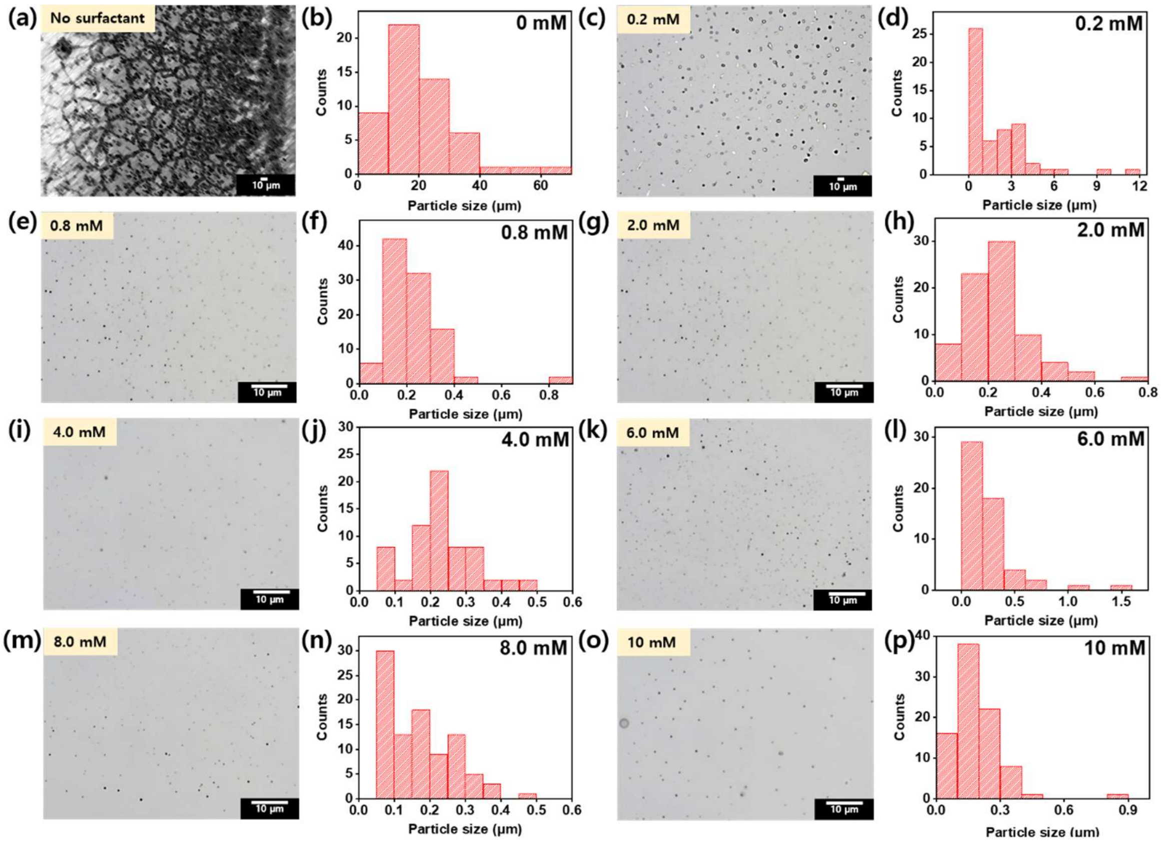

(q)

	No surfactant	0.2 mM	0.8 mM	2.0 mM	4.0 mM	6.0 mM	8.0mM	10. 0 mM
Mean (μm)	20.23	0.38	0.23	0.18	0.13	0.13	0.11	0.14
SD (μm)	14.91	2.21	0.13	0.12	0.09	0.24	0.08	0.14
Min (μm)	2.55	0.17	0.06	0.08	0.07	0.05	0.07	0.09
Max (μm)	65.32	11.18	0.87	0.71	0.47	1.53	0.46	0.87

Supplementary Figure 9. Optical images and particle distribution graphs of the CzDABNA nano-dots at (a, b) 0 mM, (c, d) 0.2 mM, (e, f) 0.8 mM, (g, h) 2 mM, (I, j) 4 mM, (k, l) 6 mM, (m, n) 8 mM, (o, p) 10 mM of TritonX-100 surfactant concentrations. The concentration of CzDABNA was kept constant at 0.01 mM; (q) Table showing their mean, standard deviation, minimum and maximum sizes.

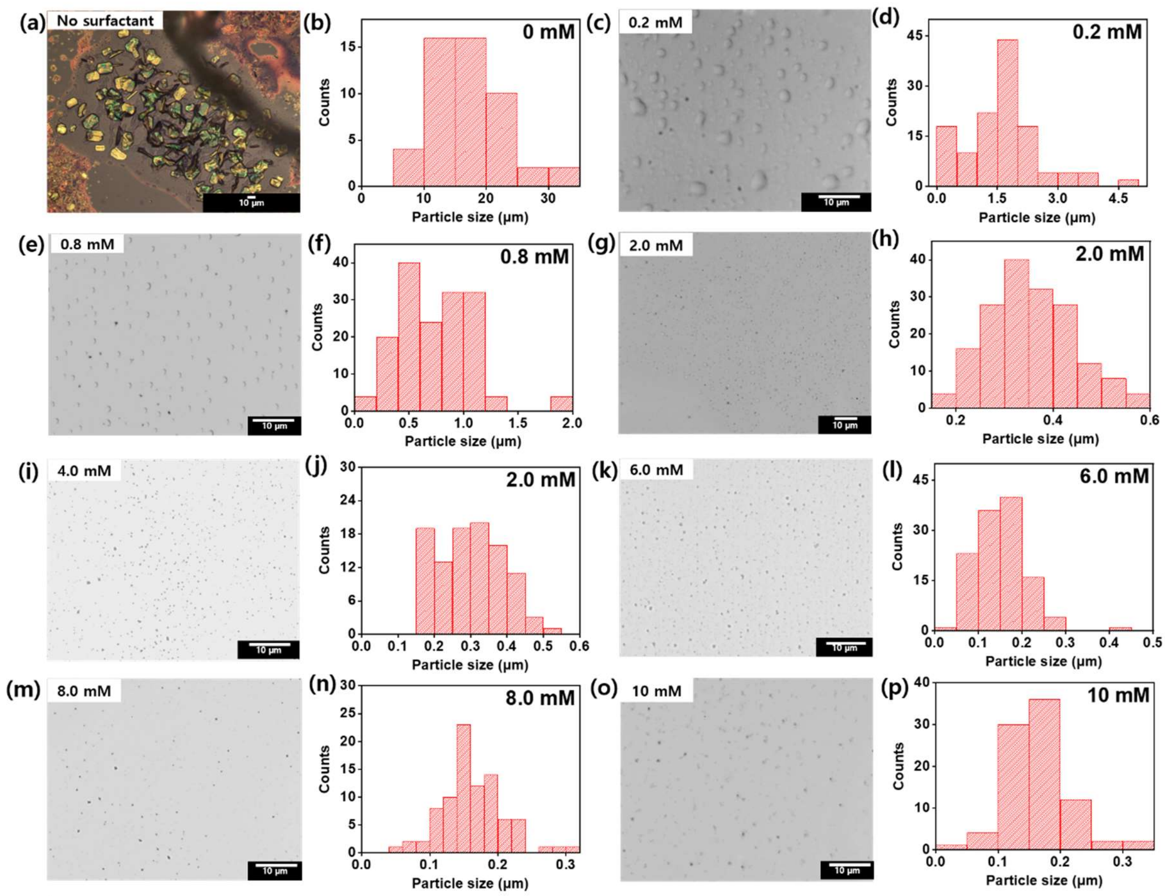

(q)

	No surfactant	0.2 mM	0.8 mM	2.0 mM	4.0 mM	6.0 mM	8.0 mM	10.0 mM
**Mean (μm)**	17.382	1.60	0.753	0.354	0.295	0.154	0.162	0.164
**SD (μm)**	4.257	0.89	0.349	0.089	0.091	0.054	0.045	0.049
**Min (μm)**	10.704	0.22	0.173	0.192	0.160	0.040	0.004	0.004
**Max (μm)**	32.343	4.59	1.872	0.595	0.540	0.420	0.303	0.343

Supplementary Figure 10. Optical images and particle distribution graphs of the 4tBuMB nano-dots at (a, b) 0 mM, (c, d) 0.2 mM, (e, f) 0.8 mM, (g, h) 2 mM, (I, j) 4 mM, (k, l) 6 mM, (m, n) 8 mM, (o, p) 10 mM of TritonX-100 surfactant concentrations. The concentration of 4tBuMB was kept constant at 0.01 mM; (q) Table showing their mean, standard deviation, minimum and maximum sizes.

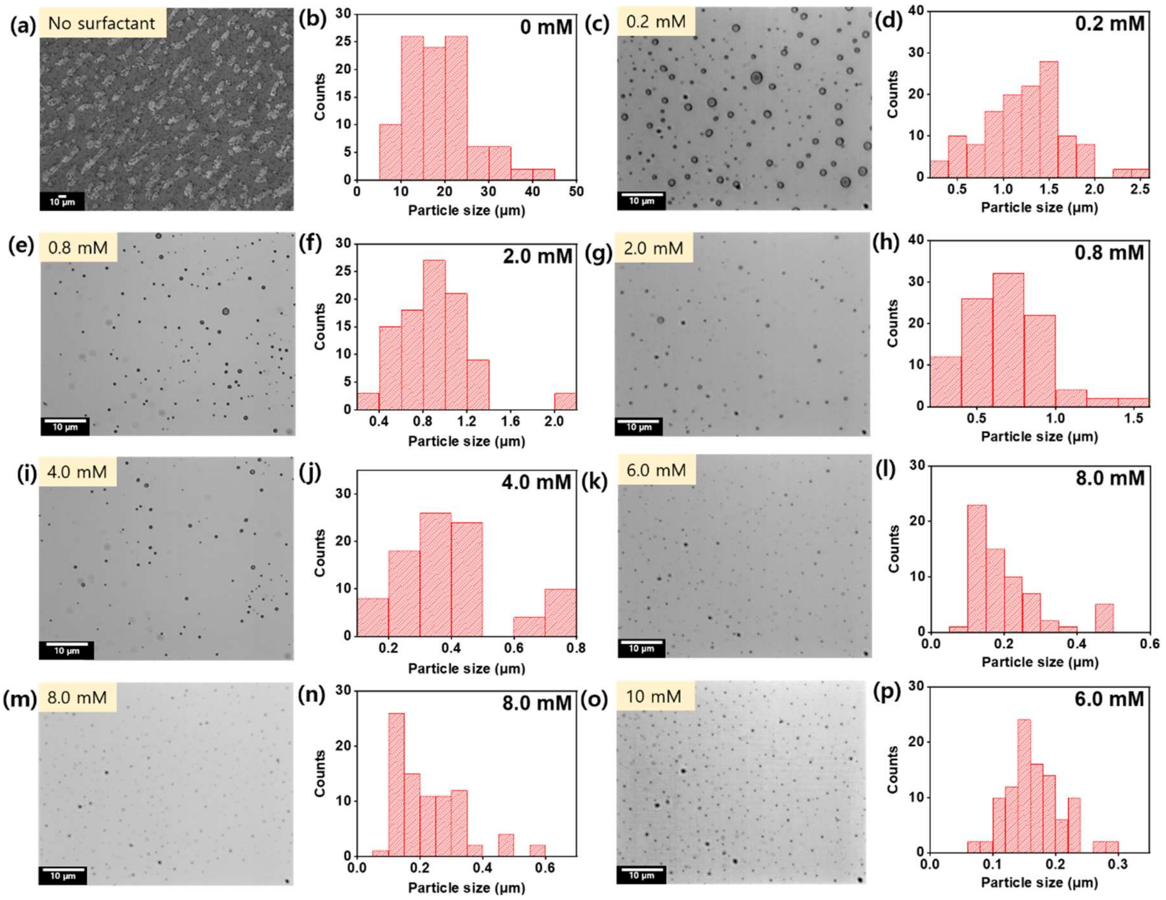

(q)

	No surfactant	0.2 mM	0.8 mM	2.0 mM	4.0 mM	6.0 mM	8.0 mM	10.0 mM
Mean (μm)	19.415	1.249	0.888	0.689	0.389	0.165	0.225	0.205
SD (μm)	7.611	0.460	0.320	0.252	0.177	0.044	0.109	0.101
Min (μm)	7.721	0.370	0.347	0.247	0.122	0.060	0.060	0.069
Max (μm)	44.646	2.548	2.001	1.459	0.787	0.285	0.559	0.499

Supplementary Figure 11. Optical images and particle distribution graphs of the TNAP nano-dots at (a, b) 0 mM, (c, d) 0.2 mM, (e, f) 0.8 mM, (g, h) 2 mM, (I, j) 4 mM, (k, l) 6 mM, (m, n) 8 mM, (o, p) 10 mM of TritonX-100 surfactant concentrations. The concentration of TNAP was kept constant at 0.01 mM; (q) Table showing their mean, standard deviation, minimum and maximum sizes.

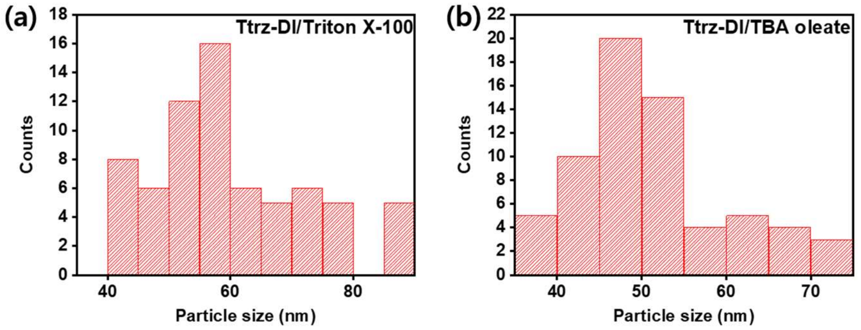

Supplementary Figure 12. Particle size distribution graph for scanning electron microscope images.

(a) Ttrz-DI with Triton X-100 surfactant, (b) Ttrz-DI with TBA oleate surfactant.

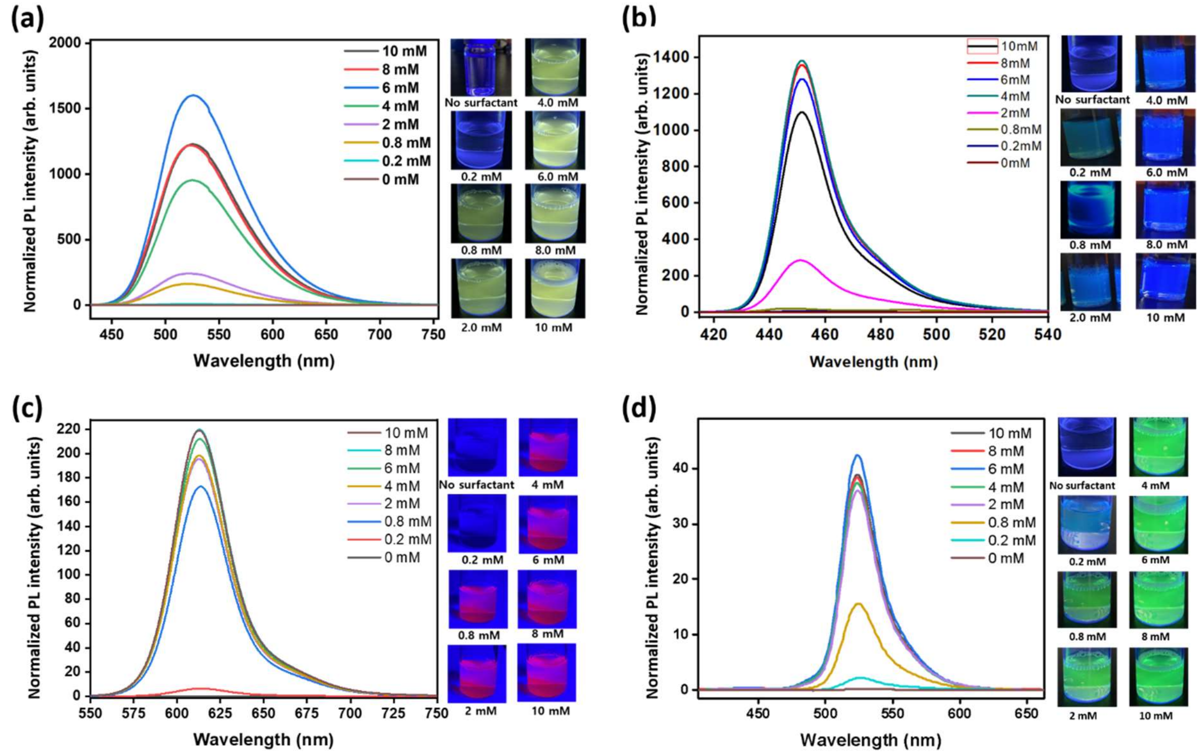

Supplementary Figure 13. Photoluminescence spectra of (a) Ttrz-DI ND, (b) CzDABNA, (c) 4tBuMB and (d) TNAP dispersions using varying concentrations of Triton X-100 surfactant where the concentration of fluorescent materials was kept constant at 0.01 mM.

Photoluminescence quantum yields (PLQY) of the organic ND dispersions and ND films were measured. We saw qualitatively in the PL measurements of Ttrz-DI ND dispersions that the PL intensity generally increased with surfactant concentration and reached a maximum at 6 mM concentration, while similar phenomena were observed in the case of CzDABNA, 4tBuMB, and TNAP ND dispersions (Supplementary Figure 13-c, d, and e). However, the PLQY values of the dispersions were lower in the case of Ttrz-DI, TNAP, and CzDABNA compared to their solutions in THF. The lower PLQY of these materials in aqueous dispersions can be attributed to their TADF emission mechanism. PLQYs of these materials rely on delayed fluorescence involving reverse intersystem crossing, rather than prompt fluorescence (singlet excited to ground state) and it is generally observed that the quantum efficiency of delayed fluorescence decreases especially in polar solvents.[3] In our study, the dispersion medium of these TADF materials was water. The electronic interactions of TADF materials with polar water molecules therefore decreased fluorescence quantum efficiency compared to solutions in organic solvents. However, in the absence of polar water molecules, solid films of the organic NDs showed higher quantum yields than their dispersions. In the case of Ttrz-DI the PLQY of the film was around 86% where its dispersion was 43%. Also, in the case of CzDABNA,

the PLQY increased slightly from 39% in dispersion to 43% in the film state. TNAP also showed similar behavior where PLQY of dispersion was 60.7% whereas, PLQY in the film state showed 84.7% quantum efficiency. This phenomenon was not observed for 4tBuMB since it is a prompt fluorescent dye compound and is not as strongly affected by interaction with polar solvent molecules; here, both the dispersion and film PLQY were close to 100%. Table 1 in the main text summarizes the PLQY and related properties for different dispersions and films.

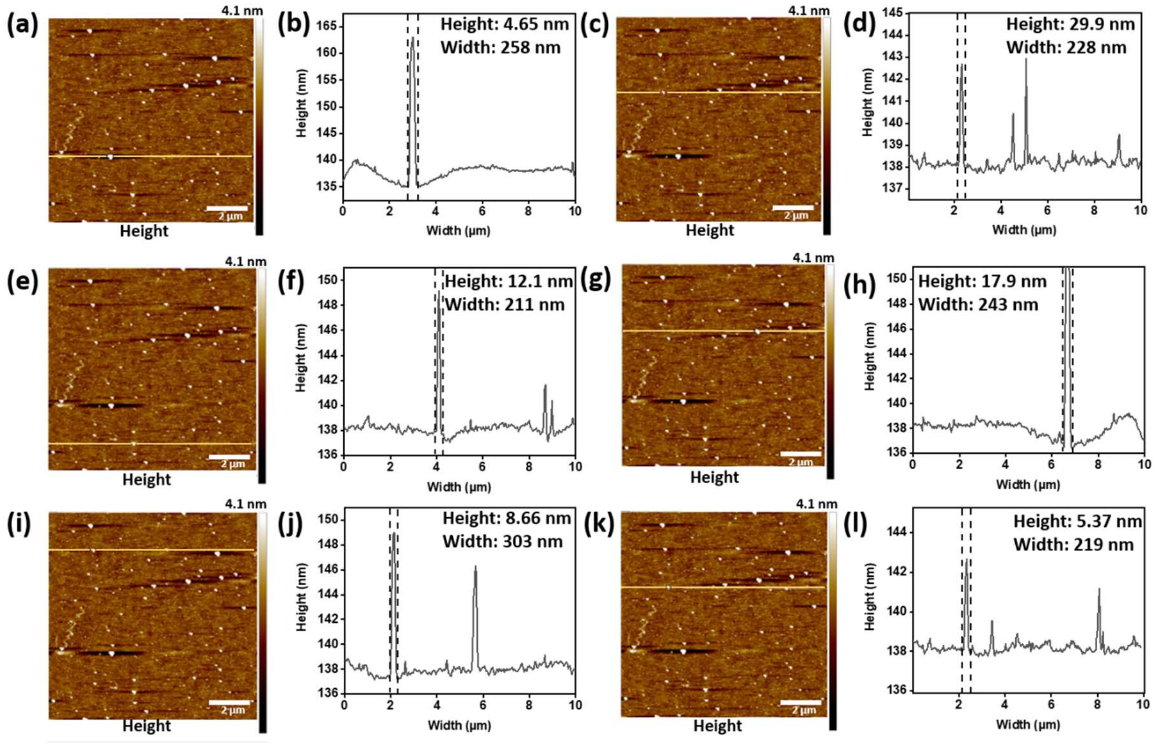

Supplementary Figure 14. Atomic force microscopy (AFM) line profile analysis of Ttrz-DI NDs. AFM topography images (a, c, e, g, i, k) of a Ttrz-DI nano-dots deposited on silicon substrates and corresponding line profile analyses (b, d, f, h, j, l) were used to determine particle diameters. The color scales correspond to heights ranging of 0 nm (black) to 4.1 nm (white) for all images.

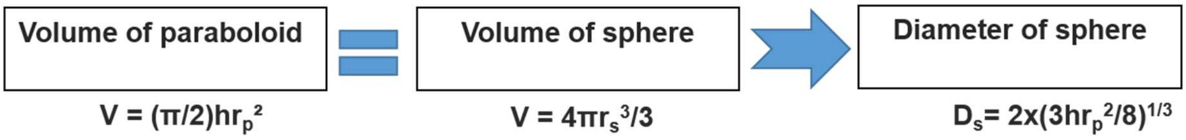

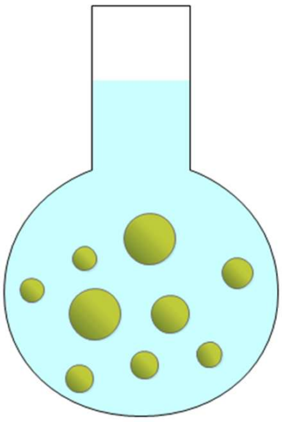

Radius of paraboloid (nm)	Height of paraboloid (nm)	Diameter of sphere (nm)
300.16	30.65	202.34
177.64	4.95	77.69
183.39	12.20	107.16
287.49	19.05	167.79
199.73	12.81	115.33
168.44	12.82	102.97
171.55	5.02	76.25
114.20	3.19	49.95
**Average diameter (nm)**		**112.44**

Supplementary Figure 15. Ttrz-DI ND size data extracted from AFM line profile analysis. The peaks obtained in the Supplementary Figure 14 were fitted as parabola, where the height of the peaks are the height of a parabola and the width of the base is the diameter. From these parameters, the volumes of paraboloid shapes seen in AFM images was calculated. Assuming that the paraboloid volume is equal to the spherical volume of the Ttrz-DI NDs when they were in dispersion, we can calculate the diameter of the nano-dots from the equation $D_s=2x(3hr_p/8)^{1/3}$ where D_s and r_s represents the diameter and radius of the NDs in dispersion while h, r_p represents the height of peak, width of base from AFM line profile in Supplementary Figure 14, respectively.

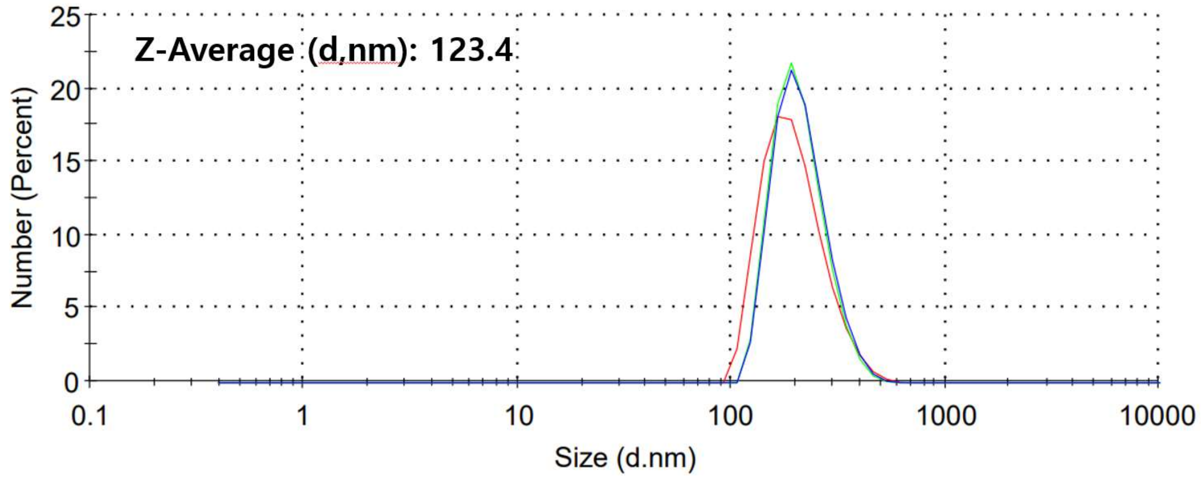

Supplementary Figure 16. Dynamic light scattering data of Ttrz-DI ND dispersions synthesized using 6 mM Triton X-100.

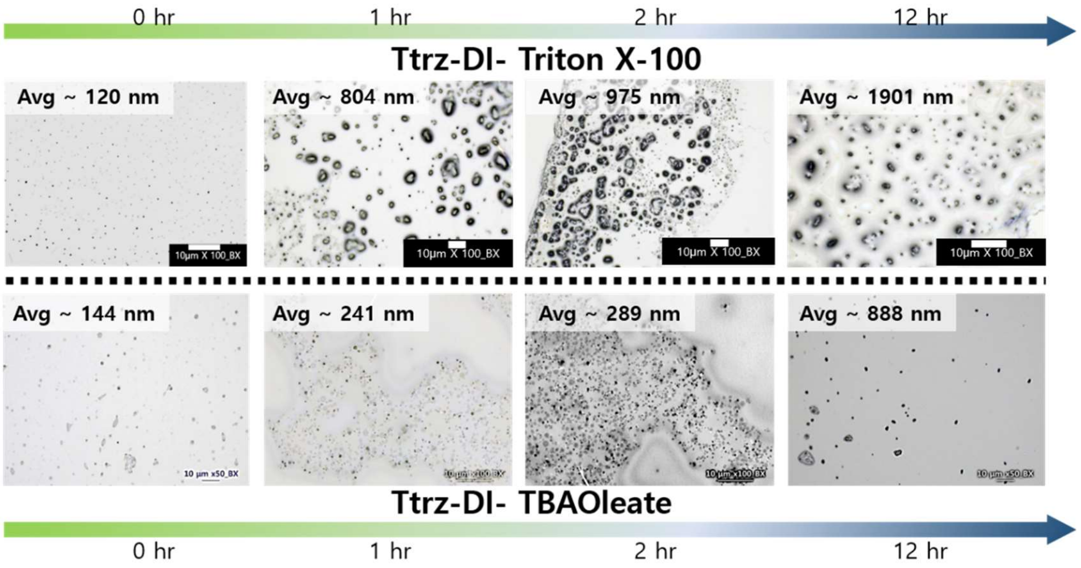

Supplementary Figure 17. Ttrz-DI ND aggregation vs time comparison using either Triton X-100 or TBAOleate surfactants at 6 mM concentration.

To characterize how Ttrz-DI ND dispersions were affected by aging, dispersions were prepared for both ionic and nonionic surfactants where the concentration of Ttrz-DI was kept at 0.01 mM. ND films were prepared by drop casting the freshly made dispersions over glass substrates. The dispersions were allowed to age for one hour and drop casted on additional glass substrates. The process was then repeated for 2 to 12 hours and the particle sizes were measured by optical microscopy. The average particle size increased from 120 nm to 1.9 μm for Triton X-100 and 144 nm to 0.8 μm for TBAOleate within 12 hours period. From the data, the aggregation rate of Ttrz-DI NDs was found greater in the case of Triton X-100 than that of TBAOleate surfactant.

.

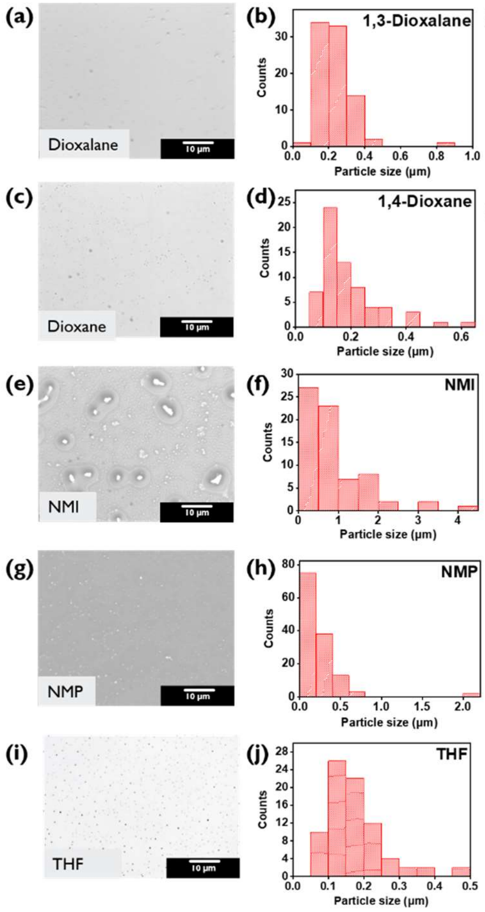

(k)

	1,3-Dioxalane	1,4-Dioxane	NMI	NMP	THF
Mean (µm)	0.24	0.19	0.86	0.26	0.12
SD (µm)	0.11	0.11	0.76	0.26	0.08
Min (µm)	0.07	0.05	0.10	0.07	0.05
Max (µm)	0.89	0.62	4.07	2.14	0.48

(l)

	PLQY (%)
1,3-Dioxalane	48.35
1,4-Dioxane	39.56
NMP	45.97
NMI	15.08
THF	43.4

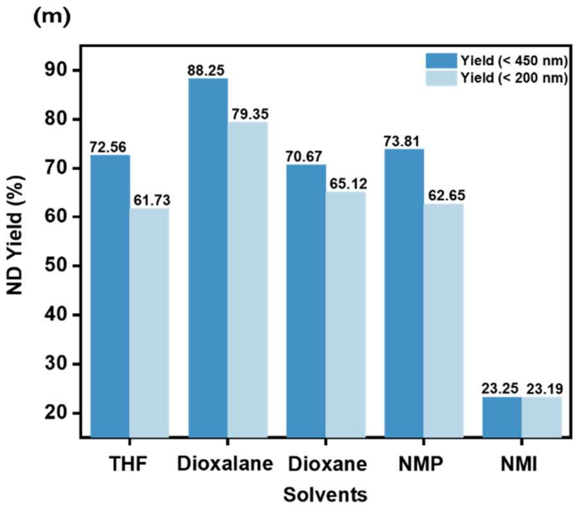

Supplementary Figure 18. Effect of different solvents on the synthesis of Ttrz-DI nano-dots. Optical microscope images and particle size distribution graphs of Ttrz-DI NDs prepared using (a, b) 1,3-dioxalane, (c, d) 1,4-dioxane, (e, f) N-methylimidazole (NMI), (g, h) N-methylpyrrolidone (NMP), (i, j) Tetrahydrofuran (THF) as solvents; (k) Table showing their mean, standard deviation, minimum and maximum sizes; (l) PLQY of the Ttrz-DI nano-dots prepared using the corresponding solvents; (m) nano-dot yields.

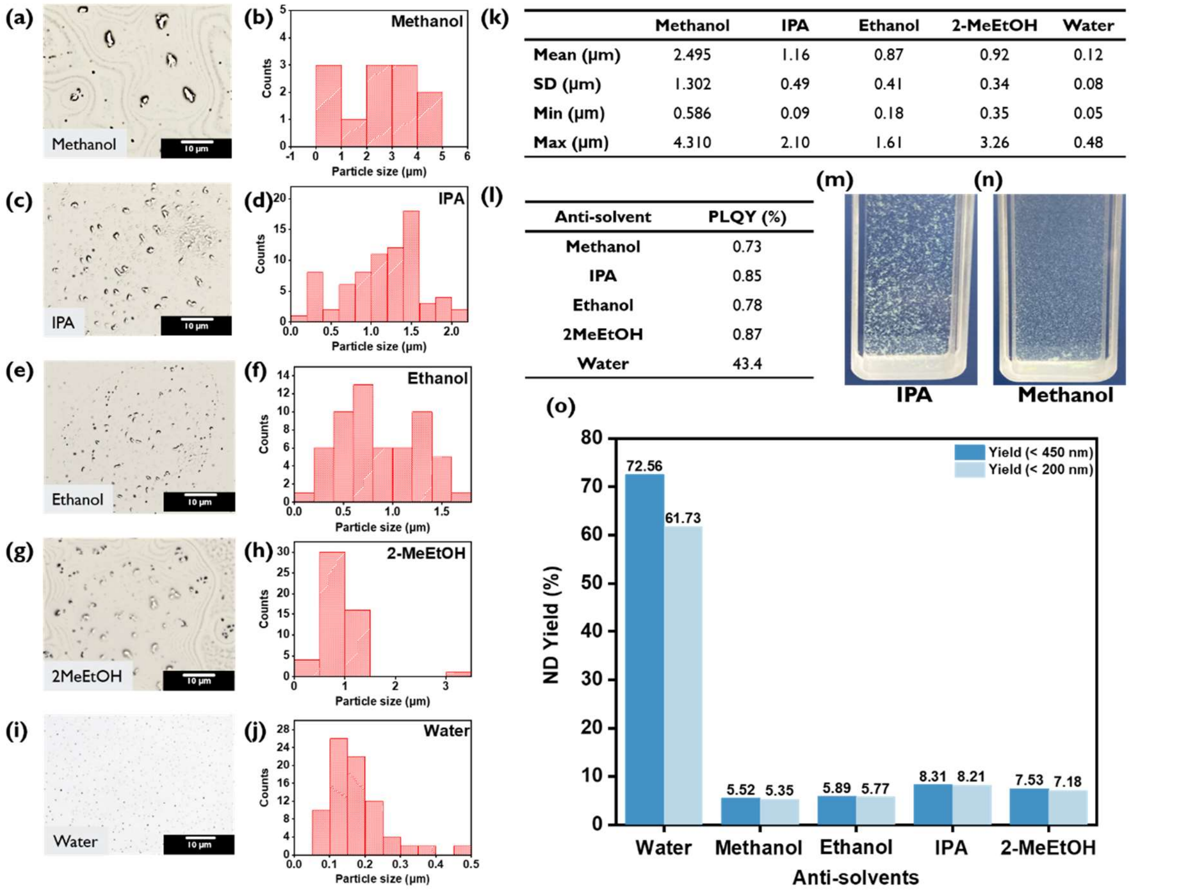

(k)

	Methanol	IPA	Ethanol	2-MeEtOH	Water
Mean (μm)	2.495	1.16	0.87	0.92	0.12
SD (μm)	1.302	0.49	0.41	0.34	0.08
Min (μm)	0.586	0.09	0.18	0.35	0.05
Max (μm)	4.310	2.10	1.61	3.26	0.48

(l)

Anti-solvent	PLQY (%)
Methanol	0.73
IPA	0.85
Ethanol	0.78
2MeEtOH	0.87
Water	43.4

Supplementary Figure 19. Effect of anti-solvents on the synthesis of Ttrz-DI nano-dots. Optical microscope images and particle size distribution graphs of Ttrz-DI NDs prepared using (a, b) methanol, (c, d) iso-propanol (IPA), (e, f) Ethanol, (g, h) 2-methoxyethanol (2-MeEtOH), and (i, j) water as an anti-solvent; (k) Table showing their mean, standard deviation, minimum and maximum sizes; (l) PLQY of the Ttrz-DI nano-dots prepared using the corresponding anti-solvents; (m) picture showing large particle size of Ttrz-DI when IPA was used as anti-solvent; (n) picture showing large particle size of Ttrz-DI when methanol was used as anti-solvent; (o) nano-dot yields.

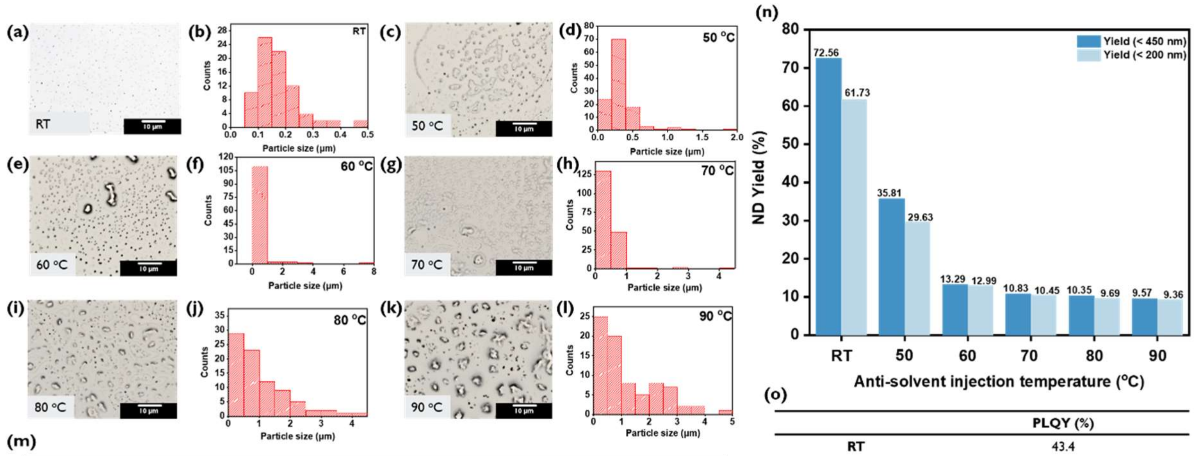

(m)

	RT (~25 °C)	50 °C	60 °C	70 °C	80 °C	90 °C
Mean (μm)	0.12	0.34	0.46	0.46	1.04	1.26
SD (μm)	0.08	0.23	0.79	0.42	0.86	1.04
Min (μm)	0.05	0.07	0.07	0.07	0.06	0.16
Max (μm)	0.48	1.83	7.28	4.07	4.31	4.74

(o)

	PLQY (%)
RT	43.4
50	23.87
60	20.47
70	16.43
80	15.68
90	12.14

Supplementary Figure 20. Effect of anti-solvent (water) injection temperature on the synthesis of Ttrz-DI nano-dots. Optical microscope image and particle size distribution graphs of Ttrz-DI NDs prepared by injecting anti-solvent water at (a, b) room temperature, (c, d) 50 °C, (e, f) 60 °C, (g, h) 70 °C, (i, j) 80 °C, and (k, l) 90 °C; (m) Table showing their mean, standard deviation, minimum and maximum sizes; (n) nano-dot yields; (o) PLQY of the Ttrz-DI nano-dots prepared by injecting into anti-solvent (water) at variable temperatures.

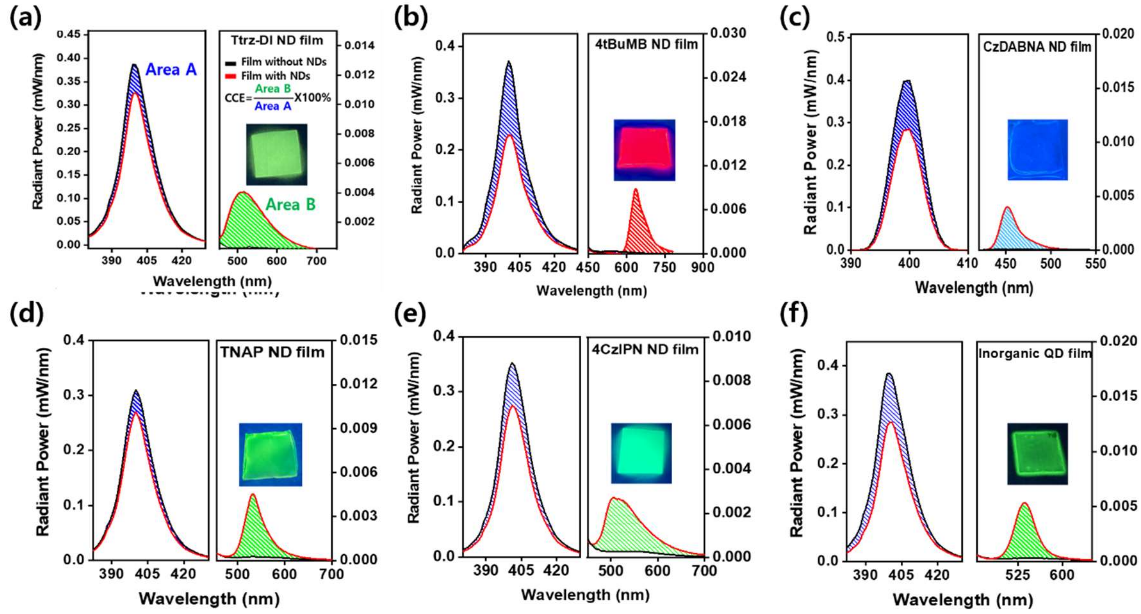

Supplementary Figure 21. Color conversion efficiency of (a) Ttrz-DI ND film, (b) 4tBuMB ND film, (c) CzDABNA ND film, (d) TNAP ND film, (e) 4CzIPN ND film, (f) Inorganic QD film. Pictures of the nano-dot films are provided in the inset. These films converted radiation with ~400 nm wavelength to their corresponding emission spectra.

Color conversion efficiency is the ability of a color conversion layer to convert the incident light emitted by a LED to a certain wavelength depending on the properties of that layer. In Supplementary Figure 21(a), the shaded area A refers to the radiant power of blue incident light absorbed by the nano-dot film and area B refers to the radiant power of green emission of the same nano-dots. From this spectrum, color conversion efficiency (CCE) can be calculated as the ratio of the radiant power of the nano-dot film to the radiant power absorbed by the nano-dot film (taken by subtracting the residual blue light area (red curve) from the total blue incident light area (black curve) without the organic nano-dots).[4] In other words, the ratio of the integrated green area B of the spectrum nano-dot film to the blue area A gives the CCE.

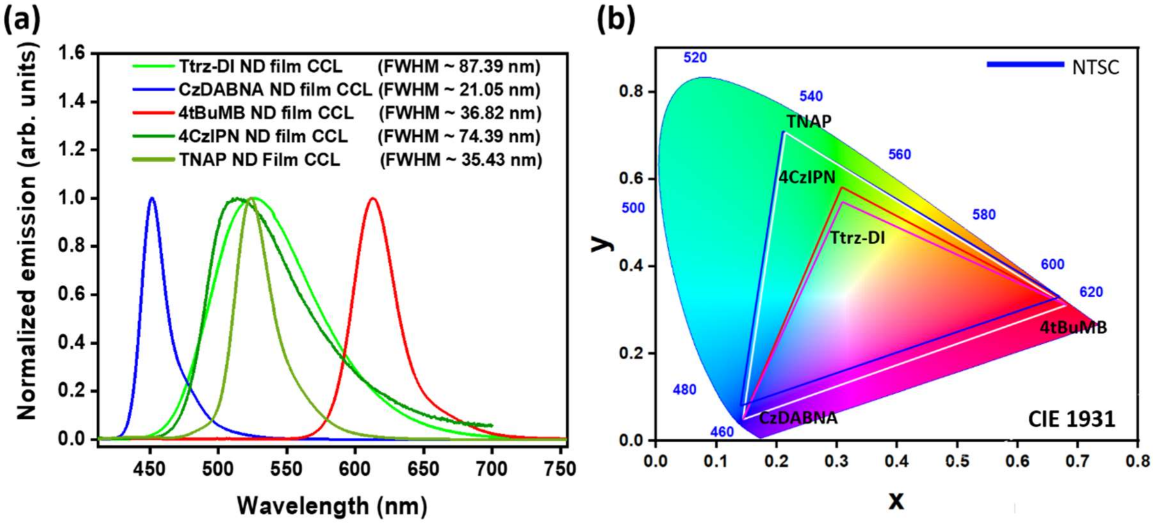

Supplementary Figure 22. (a) Full width at half maxima (FWHM) comparison of various CCL used, (b) CIE diagram of the corresponding CCLs compared to the NTSC color space

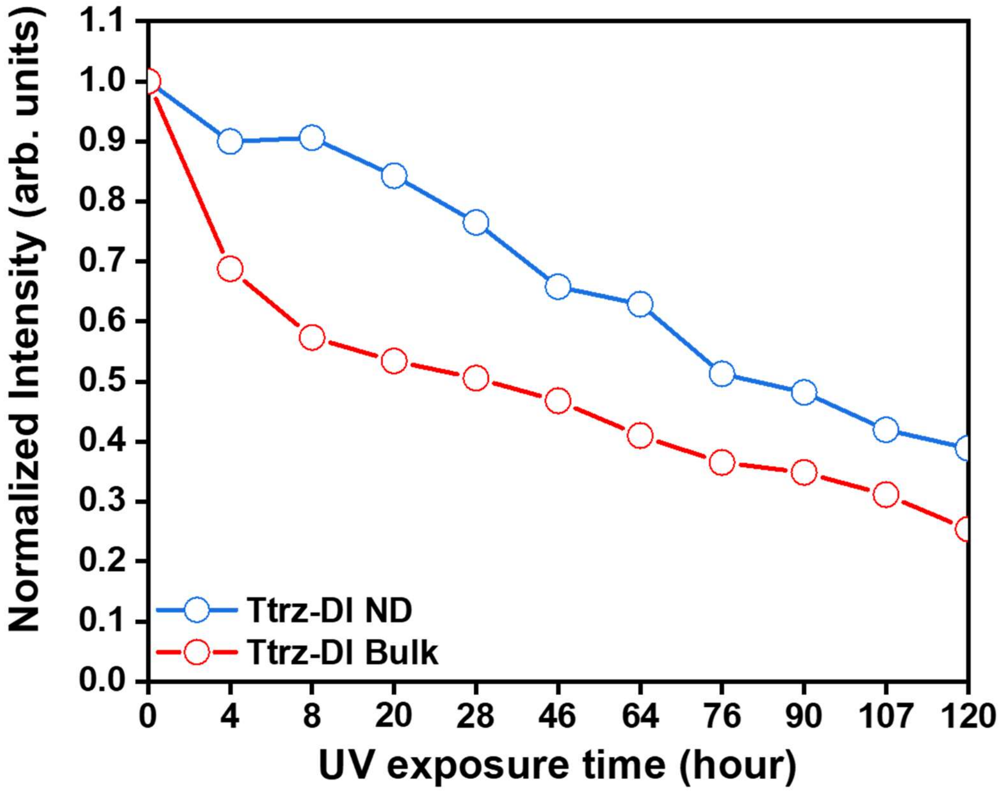

Supplementary Figure 23. Stability test comparison of Ttrz-DI ND films vs bulk Ttrz-DI film under constant UV exposure.

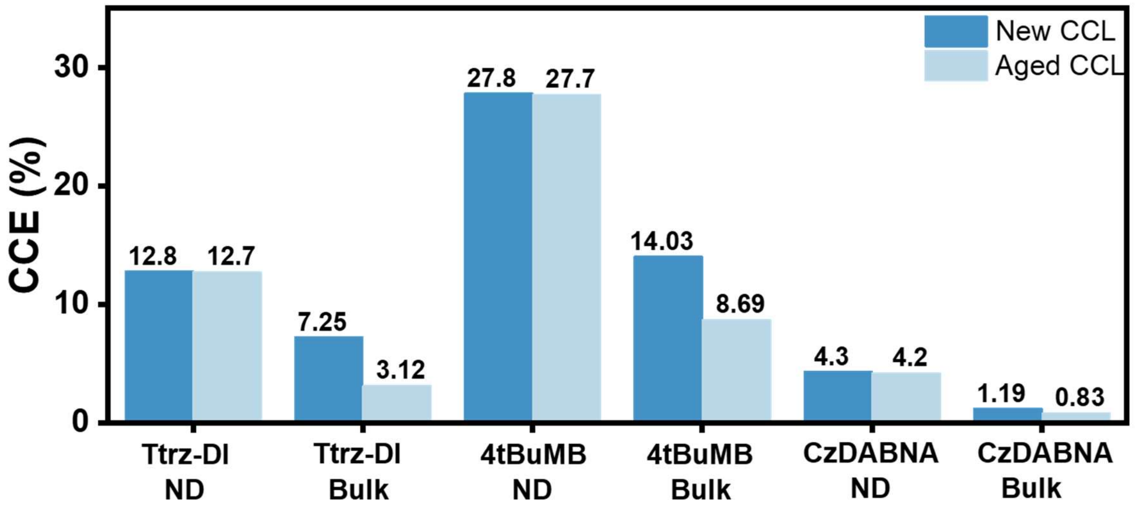

Supplementary Figure 24. Comparison of color conversion efficiency for newly fabricated CCLs and CCLs aged for one month based on nano-dot (ND) films and bulk fluorophore films.

Supplementary Table 1. Nano-dot size measurements using scanning electron microscopy.

Surfactant:	Triton X-100	TBA oleate
Mean (nm)	57.17	51.06
SD (nm)	15.89	10.36
Minimum (nm)	38.27	39.23
Maximum (nm)	89.19	71.21

The concentration of Ttrz-Di was 0.01 mM and surfactants concentration was 6 mM.

Supplementary Table 2. Comparison between ND CCLs, bulk fluorophore in PMMA CCLs and bulk fluorophore CCLs without additives.

Fluorophores	CCE (%)		
	ND films	Bulk fluorophores in PMMA	Bulk fluorophores without matrix
**Ttrz-DI (Green)**	12.80	7.25	1.46
**4tBuMB (Red)**	27.80	14.03	1.35
**CzDABNA (Blue)**	4.30	1.19	0.33
**TNAP (Green)**	31.10	14.8	2.45
**4CzIPN (Green)**	19.30	9.19	1.77

Supplementary References

1. Jung, Y. H. *et al.* A New BODIPY Material for Pure Color and Long Lifetime Red Hyperfluorescence Organic Light-Emitting Diode. *ACS Appl. Mater. Interfaces* **13**, 17882–17891 (2021).

2. Oda, S. *et al.* Carbazole-Based DABNA Analogues as Highly Efficient Thermally Activated Delayed Fluorescence Materials for Narrowband Organic Light-Emitting Diodes. *Angew.*

Chemie Int. Ed. **60**, 2882–2886 (2021).

3. Ishimatsu, R. *et al.* Solvent Effect on Thermally Activated Delayed Fluorescence by 1,2,3,5-Tetrakis(carbazol-9-yl)-4,6-dicyanobenzene. *J. Phys. Chem. A* **117**, 5607–5612 (2013).

4. Li, J. *et al.* Improvement in Color-Conversion Efficiency and Stability for Quantum-Dot-Based Light-Emitting Diodes Using a Blue Anti-Transmission Film. *Nanomaterials* vol. 8 (2018).
